# Developing an Adult Living Donor Liver Transplant Program in Western Europe: The Rotterdam Experience

**DOI:** 10.3389/ti.2025.14442

**Published:** 2025-07-10

**Authors:** Alicia Jane Chorley, Wojciech G. Polak, Khe C. K. Tran, Turkan Terkivatan, Jenny Kissler, Michail Doukas, Caroline Den Hoed, Maarten G. Thomeer, Roy Dwarkasing, Herold Metselaar, Jan N. M. Ijzermans, Robert J. Porte, Ernst Johan Kuipers, Robert C. Minnee, Markus Boehnert

**Affiliations:** ^1^ Division of HPB and Transplant Surgery, Department of Surgery, Erasmus University Medical Centre, Erasmus MC Transplant Institute, Erasmus University Medical Centre, Rotterdam, Netherlands; ^2^ Department of Anaesthesiology, Erasmus University Medical Centre, Rotterdam, Netherlands; ^3^ Department of Pathology, Erasmus University Medical Centre, Rotterdam, Netherlands; ^4^ Department of Gastroenterology and Hepatology, Erasmus MC Transplant Institute, University Medical Centre Rotterdam, Rotterdam, Netherlands; ^5^ Department of Radiology and Nuclear Medicine, Erasmus University Medical Centre, Rotterdam, Netherlands; ^6^ King Faisal Specialist Hospital and Research Centre, Organ Transplant Centre of Excellence, Riyadh, Saudi Arabia

**Keywords:** living liver donation, living donor liver transplantation (LDLT), liver transplant, liver donation, live donor liver transplant

## Abstract

Liver transplantation (LT) is curative for end stage liver disease. Expanding LT indications with limited deceased donor grafts has created organ shortages. Living donor liver transplant (LDLT) increases available organs. In 2019, we restarted our adult LDLT program. We describe our steps to create a successful LDLT program, and our outcomes. Critical steps of program development included market analysis, creation of protocols based on best care practices and a rigorous education program. Patients and donors were then actively recruited for LDLT. Outcomes were measured as morbidity (≥3 on the Clavien-Dindo grading system) and mortality. Between January 2019 and August 2024, 54 LDLT were performed. 2 (3%) donors experienced grade 3A and 7 (12%) donors experience grade 3B complications. There was no donor mortality. 22 (41%) patients were transplanted for PSC, the average MELD score was 13 (6–32). 35 (65%) patients had Roux-en-Y reconstructions. 25 (46%) complications were experienced in 22 (40%) patients, there were 2 recipient deaths. Patient and graft survival after LDLT was 97% and 97%, respectively. This paper reported the successful establishment of a LDLT program in the Netherlands. Establishing a LDLT program brings its own unique challenges, with careful planning and persistence, these challenges can be overcome.

## Graphical Abstract

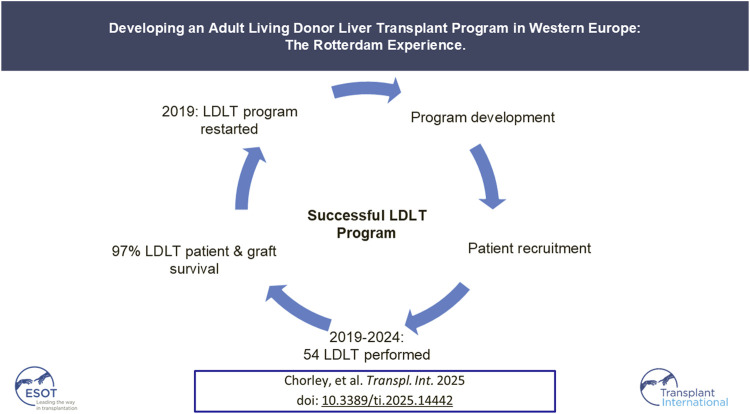

## Introduction

Liver transplantation (LT) is the only curative treatment option for end stage liver disease and selected malignancies and is a proven treatment alternative in certain metabolic diseases [1]. More than 7000 LT procedures are performed annually in Europe [1]. Since its inception, both patient and graft survival following LT have improved significantly, owing to advancements in surgical techniques, anaesthesia, immunosuppressive regimens, and the timely detection and management of complications, particularly through minimally invasive methods [2].

Initially, living donor liver transplantation (LDLT) was performed to reduce waitlist (WL) mortality in paediatric patients who faced restricted access to deceased donor organs due to size mismatches [3]. Over time, LDLT has evolved into an increasingly attractive option for adult patients and their healthcare providers, especially in locations where the demand for liver transplant exceeds the availability of deceased donor organs.

Deceased organ donation remains the predominant source for transplantation worldwide. Yet, in certain regions of the world, deceased organ donation rates remain suboptimal, often due to social, religious, logistic and cultural factors [4, 5]. This disparity has led to the growing use of LDLT, particularly in Asia and the Middle East [6]. The first successful adult LDLT’s were performed in Asia and the United States of America [7]. The favourable outcomes for both donors and recipients prompted many European LT centres to initiate their own LDLT programs during the 1990s [8]. Notably, programs in Germany and Belgium became prominent reference points for patients and their healthcare providers in Europe, contributing significantly to the field of LDLT [8]. However, due to the complexities of donor surgery, the risk of donor related complications and reports of live donor fatalities in the United States, most European transplant programs discontinued their LDLT programs [9]. LDLT is only performed in a limited number of European centres, accounting for less than 5% of total LT procedures across the continent [8].

Erasmus University Medical Centre (Erasmus MC), which performed its first LT in 1986, has since carried out more than 1,700 liver transplants. Erasmus MC first introduced LDLT in 2004, successfully performing 10 procedures between 2004 and 2011. In response to an increasing wait list mortality and the growing demand for liver transplants, Erasmus MC renewed its commitment to LDLT in 2018. This decision marked the beginning of efforts to re-establish a sustainable, successful and safe LDLT program. The aim of this article is to outline the steps undertaken to develop this LDLT program and to present the outcomes of our initial 54 LDLT procedures.

## Materials and Methods

### Program Development

#### Market Analysis and Rationale for Initiating a LDLT Program

Before launching a living donor program, it is essential to create a development plan. This plan should include market analysis to determine the feasibility and necessity of a LDLT program. According to Eurotransplant, approximately 20% of patients on the Dutch liver transplant waiting list either die or are delisted before they can receive a LT. This unmet need persisted despite advances such as machine perfusion, use of extended criteria donors, and the recent transition from an opt-in to an opt-out organ donation system in the Netherlands. These developments, while beneficial, have not sufficiently expanded the deceased organ donor pool to meet the growing demand for LT. Prior to the initiation of the LDLT program at Erasmus MC, patients were typically only eligible for LT screening and WL placement if their Model for End Stage Liver Disease (MELD) score was above 15 – except in situations where hepatocellular carcinoma (HCC) or cholangiocarcinoma was the indication for LT. This policy was based on the recognition that patients with lower MELD scores had minimal or no access to deceased donor liver transplants. As a result, cirrhotic patients with low MELD scores–such as primary sclerosing cholangitis (PSC) patients with recurrent cholangitis, or patients with metabolic liver diseases–were largely underserved. This indicated that the patient population lacking access to LT was significantly larger than the 20% who were delisted or died while on the WL. The introduction of a LDLT program would help to address this gap by offering LT options to patients with a lower MELD score already on the WL, provide access to LT for those previously deemed ineligible due to low MELD scores, and ensure timely transplantation for patients with progressive diseases such as HCC, potentially avoiding death or delisting due to disease progression.

The Netherlands presents a favourable environment for launching a LDLT program. As a multicultural society, it supports a diverse patient and donor base. Importantly, the Dutch legal framework permits all forms of living donation–related directed, unrelated directed, and unrelated undirected donation–unlike some other European countries where regulations are more restrictive. Furthermore, the success of large living kidney donor programs in The Netherlands indicated both public awareness and acceptance of the concept of living donation. These factors combined suggest a receptive donor population and a clear, unmet medical need among LT recipients. This market analysis strongly supported the initiation of a LDLT program as both a necessary and viable addition to liver transplant services in the Netherlands.

#### Program Development and Resource Allocation for LDLT Initiative

Following the completion of the market analysis, the next critical phase in launching a LDLT program involved identifying structural, human and procedural requirements. These would be necessary for successful implementation and long-term sustainability of the LDLT program. To ensure that the program would be well supported, all relevant multidisciplinary stakeholders were invited to share their concerns and perspectives, this collaborative approach allowed the LDLT program to be integrated into the broader LT program. Key staff members were recruited to establish the programs’ foundation, including the recruitment of an experienced LDLT surgeon and LDLT nurse coordinator. These individuals were tasked not only with the establishment and day-to-day running of the program, but also with ensuring its continuity through training of existing medical personnel involved in the deceased liver transplant program.

Institutional support for the LDLT program was both early and robust. The LDLT initiative received endorsement from the board of directors, chief executive officer and leadership within both the liver transplant surgery and hepatology departments. In accordance with international ethical guidelines and standards, an independent live donor advocate was also appointed to protect the interests and autonomy of all potential donors throughout the evaluation and donor screening process [10].

A comprehensive workflow analysis of the existing deceased donor LT program was undertaken to identify similarities and gaps. Based on this assessment, strategic recruitment efforts were undertaken to expand the multidisciplinary team. This included two specialized radiologists proficient in high resolution MRCP and CT scans as well as radiology technicians trained in liver volumetry techniques. To optimize patient outcomes and perioperative care, an intensivist was brought onboard to provide specialist oversight during the ICU stay for the donor and recipient, serving as the primary liaison during their respective ICU stays. A social worker and psychologist were integrated into LDLT program to support potential donors during the screening process, ensuring holistic psychosocial evaluation and preparation. These healthcare professionals would also be available to support donors if needed after donation as well. Table 1 details the individuals involved in each stage of donor screening and follow-up.

**TABLE 1:** Manpower involved in donor screening and follow up.

Screening phase	Nurse practitioner Live donor surgeon Social worker Psychologist Anaesthesiologist Radiologist Cardiologist
Peri-operative phase	Live donor surgeon Liver transplant surgeon Nurse practitioner Nurses Anaesthesiologist
After care phase	Nurse practitioner Live donor surgeon Social worker and psychologist (as needed)

In anticipation of clinical activities, all necessary surgical and supportive equipment–including specialised instruments, foot pump devices, incentive spirometers–was procured prior to the enrolment of patients and donors in the program. This preparatory phase ensured operational readiness and underscored the institutions commitment to delivering safe, ethical and sustainable LDLT program.

#### Education and Capacity Building

A comprehensive education and training program was implemented to ensure all health professionals involved in the care of potential living liver donors and recipients possessed the required knowledge, clinical competencies and ethical awareness to manage this complex patient population effectively. The education program was designed to foster deep understanding of the principles, procedures, ethics and psychological dimensions unique to a LDLT program.

All staff members who would have contact with donors and recipients were targeted for training and education. Specialised education and training sessions were delivered to nursing staff in the operation room (OR), intensive care unit (ICU) outpatient clinic and inpatient care settings. This approach aimed to standardise clinical care pathways, enhance communication between teams and ensures both donors and recipients received consistent, high-quality care throughout the entire donation and transplant process.

#### Workflows and Protocol Development

A critical component in the establishment of a LDLT program was the development and implementation of standardised workflows and protocols to guide the evaluation and clinical management of both potential living liver donors and potential LDLT recipients. These protocols clearly defined the eligibility parameters, indications and contraindications for donation and LDLT, as well as specific clinical, ethical and psychosocial considerations to be evaluated throughout the assessment process. Clinical pathways were established to outline the specific day-to-day care of post donation donors and LDLT recipients. These pathways were designed to standardize care delivery, facilitate multidisciplinary coordination, and ensure that each patient received high quality, patient centred treatment in all clinical settings. Given Erasmus MC status as an international training centre, all procedural documents and clinical materials were available in both Dutch and English. Additionally, patient resources–including detailed, user-friendly information booklets for both donors and recipients–were produced to as a tool to enhance patient understanding and program transparency. These booklets outlined the structure of the LDLT program, provided educational information about LDLT and donation, and included information on national resources available within the Netherlands such as the Dutch Transplant Foundation. The financial impact of living donation is not to be underestimated; therefore, information on the financial impacts and subsidies available for donors was also included in education materials. The risks, potential complications, and long-term implications of both donation and transplantation were addressed in detail as part of the pre-screening and consent process. Although written consent prior to medical procedures is not a legal requirement in the Netherlands, the unique complexity of LDLT and the interdependence of the donor and recipient procedures prompted the adoption of a formal written informed consent process for both donors and recipients. This decision reflects the program’s commitment to ethical standards, respect for patient autonomy, and the safeguarding of all individuals involved in the donation and transplant process.

#### Risk Assessment and Mitigation Planning

Prior to the initiation of clinical activity, a comprehensive risk assessment was performed to systematically identify, evaluate and address potential pitfalls and complications that could occur once the living donor program was functioning at Erasmus MC. This evaluation involved extensive consultation with all relevant stakeholders, including surgery, hepatology, anaesthesiology, intensive care, radiology, psychosocial, and administrative teams. Each discipline was invited to provide their input on potential risks within their domain of expertise using the risk assessment tool RISKID. Participants could anonymously enter potential risks and hazards from their perspective into this system. Once potential hazards had been identified, these risks could be evaluated based on the likelihood of occurrence, who might be harmed and how severe the consequences of the event would be. All findings were recorded, and actions were implemented based on the risk levels. Existing protocols and procedures were rigorously reviewed to confirm that anticipated complications—both routine and exceptional—had been adequately addressed prior to clinical activity. Finally, the risk assessment and updated protocols were updated and reviewed by all stakeholders participating in LDLT. This risk assessment is updated periodically in line with institutional requirements, after near miss incidents, or when new processes are implemented.

To further enhance preparedness, a Crisis Management Plan was developed and documented. This plan outlined clear, stepwise procedures for responding to major complications in living donors, including intraoperative adverse events and severe postoperative morbidity. A formal crisis response statement was also prepared, highlighting Erasmus MC’s institutional commitment to transparency, ethical accountability, and donor protection.

This risk assessment process reinforced a culture of safety, readiness, and continuous quality improvement as foundational principles of the LDLT program.

### Implementation

#### Patient Recruitment and Candidate Selection

Following the establishment of clear eligibility criteria for both living liver donors and LDLT recipients, the program progressed to the active recruitment of potential donors and recipients. This phase was designed to identify suitable donor and recipient pairs, while maintaining safety, transparency, and ethical integrity. In October 2018, an initial cohort of 20 patients was selected from the deceased donor WL based on a comprehensive review of their diagnosis, previous medical and surgical history, MELD score and Child-Pugh score. These 20 patients were identified as potentially appropriate candidates for LDLT, given their clinical profiles and likelihood of limited access to deceased donor grafts. Each of the selected patients was invited to the outpatient clinic for a detailed consultation, where their own hepatologist and the LDLT surgeon explained the concept of LDLT including the risks, benefits, possible complications, and donor criteria. This personalized approach ensured patients were given the opportunity to make informed decisions, based on accurate information. Subsequently, a structured and ongoing LDLT screening process was also initiated, where all patients currently on the deceased donor waiting list as well as all new referrals to the transplant centre were reviewed on a weekly by the LDLT nurse coordinator. This continuous review process enabled the early identification of potential new candidates for LDLT. This strategic and patient-centred approach to recruitment allowed for early identification of donor-recipient pairs and contributed to the broader goals of expanding the LDLT program and access to LT.

#### Donor Selection and Evaluation

Donor selection and evaluation are the ethical and clinical cornerstones of any LDLT program. The screening process must be methodical, evidence based and sufficiently stringent to exclude any individual for whom the donation procedure poses an elevated or unacceptable risk. Furthermore, donor evaluation must prioritise the long-term health, safety and quality of life, of the donor, ensuring that no compromises are made in pursuit of recipient benefit. To uphold these principles, the donor evaluation process at Erasmus MC was designed to proceed in a stepwise manner, with the explicit goal of identifying and excluding unsuitable donors as early as possible in the screening process. This approach minimizes unnecessary testing and reduces the physical and psychological burden on potential donors.

The donor selection criteria included individuals who were related, unrelated directed and unrelated undirected to their recipient, aged between 18 and 55 years, with a body mass index (BMI) of less than 30, blood group compatible with the recipient, and to ensure the absence of any major medical history or surgical procedures (Table 2). Donors would be accepted if they voluntarily came forward to donate and were physically and psychologically fit to provide informed consent. Potential donors must have a clear ability to understand the risks, benefits and long-term complications associated with donation. These criteria were applied uniformly across all donor types (related directed, unrelated directed and unrelated undirected donors) to maintain consistency and safeguard donor welfare.

**TABLE 2:** Donor and recipient selection criteria.

Donor suitability criteria	Recipient suitability criteria
18–55 years	Indications following international criteria
BMI <30 kg/m^2^	Formally listed with Eurotransplant
Remnant liver volume ≥30%	No re transplantation, no expected arterial/venous jump grafts
Blood group compatible with recipient	
Psychologically and physically healthy, no previous major surgery	

BMI: Body Mass Index.

The structured and ethical approach to donor evaluation reflects the program’s commitment to the principle of *primum non nocere*—first, do no harm—while enabling access LDLT through safe and responsible living donation.

Beyond ensuring medical and surgical suitability, the overarching goal of any living donor program is the steadfast commitment to donor safety, autonomy and wellbeing. At Erasmus MC, donor voluntariness is regarded as a fundamental prerequisite for participation in the screening process and subsequent donation. It is imperative that all potential donors engage in the donation process free from coercion, external pressure, or undue influence of any kind. In alignment with international ethical standards, Erasmus MC does not actively solicit or recruit living donors. The presence of any form of coercion—be it emotional, social, or financial—automatically makes a potential donor unsuitable for living liver donation. Financial incentives or indirect compensation are explicitly prohibited in the LDLT program at Erasmus MC, any indication of incentives or compensation for organ donation results in an immediate discontinuation of the evaluation process. To further safeguard donor autonomy, a donor advocate is integrated into the live donor team to provide additional oversight as needed. Additionally, all potential living donors are explicitly informed–during a private consultation with the LDLT surgeon—that they may withdraw from the process at any point without the need to justify their decision to the LDLT team or the recipient. Any withdrawal from the donation process can be framed as a medical contraindication, thereby protecting the donor from social or familial repercussions.

All potential liver donors were self-referred, no referral is needed from another health professional to begin the donor screening process. The majority of living liver donors are family members or close family friends of the intended recipient. Potential donors initially contact the LDLT nurse coordinator directly, where donors were pre-screened for suitability in terms of age, BMI, blood group compatibility, previous medical/surgical history (Table 2). Potential donors who met the initial selection criteria were invited for a structured intake and information session with a qualified LDLT surgeon and LDLT nurse coordinator in the outpatient clinic. This session provided potential donors with a detailed overview of the donation process, surgery, associated risks, and the expected recovery period. Potential donors then underwent extensive blood testing, which included but was not limited to blood group typing, renal and liver function, haematological investigations, extensive coagulopathy screening, virology, and infectious screening. Donors with satisfactory blood test results who expressed a willingness to proceed with donor screening, were then planned for the next screening phase. An interview with social worker determined if the potential donor had adequate support systems in place to manage the pre, peri, and post-operative periods. A comprehensive psychological evaluation with a psychologist assessed the potential donors’ motivation for donation, expectations, and current relationship with recipient, coping mechanisms, and any previous life events or psychiatric history which may affect decision making, delay or inhibit recovery after donation. To ensure the donor could safely undergo anaesthesia, lung function tests, a chest X-ray and electrocardiogram were performed. Advanced radiological imaging played a pivotal role in determining the anatomical and technical feasibility of living donation and transplant. A four phase CT scan was performed to confirm the absence of focal liver lesions, abnormal pathology in the abdomen, and to assess the liver quality, venous, and arterial anatomy. Liver volumetry was performed on the CT images to calculate segmental liver volumes using specialised volumetric software. All donors underwent an MRCP to determine biliary anatomy and rule out structural anomalies. Donors with an estimated remnant liver volume of less than 30% were excluded from donation due to unacceptable risk. Additionally, the estimated graft recipient weight ratio was required to exceed >0.7 to ensure adequate liver function post-transplant. Potential donors who satisfied the criteria in the first two phases of the screening process were then referred to anaesthesia for clearance, and an echocardiogram was performed. The final stage of evaluation included a liver biopsy, which allowed for assessment of steatosis, fibrosis, inflammation, iron overload or alpha-1 antitrypsin in the liver–any of which could be a contraindication for donation. Only after successful completion of all screening phases and multidisciplinary team review were potential donors formally approved to undergo living liver donation surgery.

In line with ethical and clinical best practices, any incidental findings during screening—such as previously undiagnosed medical conditions—triggered referral to the appropriate specialists within Erasmus MC for further evaluation and management.

#### LDLT Recipient Evaluation

Prior to being considered for LDLT, all potential recipients must undergo evaluation for LT in accordance with the national liver transplant screening protocol and be placed on the Eurotransplant waiting list [11, 12]. This ensures that LDLT candidates are first deemed appropriate for LT based on national and international standards. To further optimize outcomes in the early phase of the LDLT program, specific inclusion, and exclusion criteria for LDLT were established. In the initial phase of the program, patients anticipated to present significant surgical complexity–re transplantations, polycystic liver disease patients, and patients with complete portal vein thrombosis–were excluded as candidates for LDLT (Table 2).

Potential LT candidates are referred by hepatologists in peripheral hospitals to Erasmus MC when their MELD score exceeds 15. Direct referrals are also accepted for patients with hepatocellular carcinoma (HCC) or cholangiocarcinoma, given the time sensitive nature of these indications. Upon referral, new potential recipients who meet the criteria for LDLT are introduced to the LDLT program during their first visit by the LDLT nurse coordinator, who provides detailed information about the program including LDLT risks and benefits, donor criteria and donor screening processes (Table 3). Potential recipients who meet criteria for LT then proceed to a 2–3 days inpatient evaluation at Erasmus MC. Recipient evaluation includes, but is not limited to CT and Magnetic resonance imaging (MRI) scans, echocardiogram and electrocardiogram (ECG), appointments with social worker, anesthesia, infectious diseases specialist, liver transplant surgeon, dentist, and ear nose throat specialist, bone density scan, gastroscopy and colonoscopy, lung function tests, chest X-ray, and blood tests. At the completion of screening, each potential recipient is presented in a multidisciplinary team meeting consisting of anesthesiologists, hepatologists, social work, and transplant surgeons. This team collaboratively determines the patients’ suitability for LT based on medical, surgical, psychosocial, and logistical factors. Recipients deemed eligible for LT are then placed on the Eurotransplant liver transplant waiting list, with any potential living liver donors evaluated in parallel where appropriate.

**TABLE 3:** Donor screening phases.

Donor screening
Phase 1: Intake interview, information conversation, blood testing
Phase 2: Social work and psychological screening, Chest X-ray, ECG, lung function tests, CT scan, MRCP
Phase 3: Surgical clearance, anaesthesia clearance, echocardiogram
Phase 4: Liver biopsy

ECG, electrocardiogram; CT scan, computerized tomography scan; MRCP, Magnetic resonance cholangiopancreatography.

#### Donor Surgery

Surgery was performed under general anesthesia. A central venous catheter, arterial line urinary catheter and peripheral intravenous cannula were placed for safety and hemodynamic control at the beginning of and during the donor surgery. An upper midline incision was used with the Thompson Retractor^®^. After inspection and palpation of the liver, the right or left lobe of the liver is fully mobilized. The gallbladder is mobilized off the liver bed and an intraoperative cholangiogram is performed to verify biliary anatomy. Depending on a right or left liver lobe donation, the right or left hepatic artery and portal vein are dissected and encircled. The right hepatic vein is encircled with an umbilical tape running between with liver and IVC, which is used during the parenchymal transection. The transection line in our center is on the right side of the middle hepatic vein keeping the latter always to the left liver lobe. Extending to the mid-point of the gallbladder fossa, is marked and liver dissection is performed with Cavitron Ultrasonic Surgical Aspirator (CUSA). The liver graft was procured and flushed with University Wisconsin solution via the right/left portal vein and right/left hepatic artery. A Jackson Pratt (JP) drain was placed at the end of surgery, with the tip of the drain next to the resected liver. After surgery was completed, trans-abdominal plain blocks or rectal sheath catheters were placed by anesthesia for pain relief.

#### Recipient Surgery

Surgery was performed under general anesthesia. Peripheral intravenous catheter, arterial line, central venous catheter, pulmonary artery catheter was placed for monitoring and hemodynamic control; transesophageal echocardiogram monitoring was performed during surgery where indicated. A reversed L-shape incision was used. After mobilization of the left and right lobes, hepatica artery, portal vein, and bile duct were dissected, and divided as high as possible. After the hepatectomy, the hepatic vein reconstruction was performed with polene 5.0. The portal vein was anastomosed using prolene 6.0 or 7.0. After portal-venous reperfusion, the hepatic artery was reconstructed with interrupted prolene 8.0 sutures. An intraoperative Doppler ultrasound of the liver was performed to confirm patency of all blood vessels. Duct-to-duct anastomosis or Roux-en-Y anastomosis was employed for the biliary reconstruction with interrupted PDS 7.0 sutures. Two abdominal drains were placed intra operatively in the recipient: one in the liver hilum and the second behind the liver lobe.

#### Post-Operative Management

Initially, both living liver donors and recipients were admitted to the Intensive care unit (ICU) for overnight monitoring following surgery. However, in response to the increased demand for ICU resources during the COVID-19 pandemic, a revised protocol was implemented. Under this new protocol, donors are now admitted to the post anesthesia care unit (PACU) for the first postoperative night before returning to the surgical ward on day 1 to continue recovery.

The focus of donor post-operative care is ensuring donor safety and comfort. Postoperative management is initially focused on adequate pain control, prompt mobilization, correction of electrolyte imbalances due to the rapid regeneration of liver tissue and prevention of complications. Donors are extubated in the operating room prior to transfer to the PACU. A mild elevation in lactate levels is common immediately postoperatively which is routinely managed through aggressive fluid resuscitation. Pain management is multifaceted. A patient-controlled analgesia pump provides continuous and bolus breakthrough pain management which is typically kept for 2–3 days–dosages are reduced daily before switching to oral pain relief when patient-controlled analgesia is ceased. Post-operative pain is also managed with transverse abdominis plane (TAP) blocks or rectal sheath catheters–these are refilled with a local anesthesia agent such as ropivacaine every 8 h and provides targeted pain relief for 3–4 days postoperatively.

After donation surgery, all living donors undergo daily monitoring of key clinical, biochemical parameters to ensure the prevention, (early) detection and management of complications. Laboratory tests are performed to assess liver function, renal function, electrolytes, coagulation, and infection parameters. In addition to laboratory monitoring, an abdominal ultrasound is performed on day 0 and day 5 to ensure vascular patency (hepatic artery, portal vein and hepatic veins) and to identify the presence of any peri-hepatic fluid collections. Prophylactic antibiotic therapy is administered until the abdominal drain is removed, in line with the infection prevention protocol in our LDLT program. Early mobilization is a key component of postoperative care and begins on post-operative day 1 facilitated by a physiotherapy team. Physical activity is progressively increased each day to support circulation, pulmonary function, and overall recovery. Donors are typically discharged between postoperative day 5 and 7, depending on their clinical recovery. Following discharge, donors are then followed intensively in the outpatient setting for the first year after donation. After completing the first postoperative year without complications, donors have the option to return to Erasmus MC yearly for an appointment or complete blood testing via their general practitioner followed by a remote consultation with the LDLT nurse coordinator. This follow-up protocol ensures comprehensive short- and long-term monitoring of donor health and underscores Erasmus MC’s commitment to donor safety and wellbeing.

Following LDLT, recipients are typically admitted to the ICU for 2–3 days for close observation. In cases where surgery proceeds uneventfully, recipients may be extubated on the OR table. Otherwise, recipients are extubated within 8–12 h postoperatively, once clinically stable. Postoperative care is delivered by a multidisciplinary team including the attending and consultant hepatologist and nurse practitioner, as well as live donor nurse practitioner/LDLT surgeons. This collaborative approach ensures continuity of care and supports the early identification and management of potential complications. To monitor for vascular complications, daily liver ultrasounds are performed from postoperative day 0 to day 7. Recipients receive a standardized immunosuppression regimen consisting of induction with methylprednisolone and basiliximab that is given day 0 and day 4 and maintenance with mycophenolic acid and prednisone from D0 followed by tacrolimus beginning on day 5 post-operatively. Once two adequate trough levels of tacrolimus have been achieved, mycophenolic acid is discontinued. Prednisone is tapered over a 3–6-month period, depending on the clinical course. Recipients also receive prophylactic antibiotics until the drains are removed.

Most recipients are discharged from the hospital within 14 days following LDLT, assuming a stable recovery without significant complications. After discharge, patients are closely monitored in the outpatient with regular laboratory investigations, imaging, and appointments with hepatologists and nurse practitioners. Immunosuppressant levels and compliance are monitored to ensure optimum graft function and long-term success.

The study was reviewed and approved by the Medical Ethics Review Committee of the Erasmus MC (MEC-2023-0774). The donors and patients provided written informed consent to participate in this study.

## Results

**FIGURE 1:**
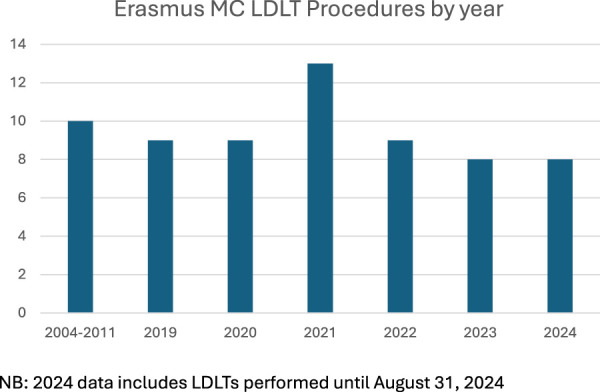
LDLT procedures performed at Erasmus MC, by year.

Outcomes from LDLT procedures performed between January 2019 and August 2024 were included in this analysis. A total of three donor procedures were aborted intra-operatively due to the identification of abnormal biliary anatomy, which was previously undetected on pre-operative imaging. To ensure accuracy and consistency of results, these donors and their corresponding recipients have been excluded from the reported data. In the final quarter of 2020 and the first half of 2021, our ability to perform LDLT was significantly impacted by the COVID-19 pandemic, which placed considerable strain on hospital resources. Specifically, operating room availability, ICU bed capacity, and admission scheduling were constrained due to prioritization of critical care for COVID-19 patients (Figure 1). Despite these challenges, the program demonstrated resilience and adaptability, with a subsequent rebound in case numbers as hospital operations normalized.

### Donor Outcomes

Donor characteristics are reported in Table 4. Most donors were related to their recipients, with 36 donors (66%) being female. The median donor age was 33 years (range 18–58 years), and the mean BMI was 27 kg/m^2^ (range 17–31 kg/m^2^). Fifty-two right lobe donations took place, as well as two left lobe donations and two domino LDLT. The median length of hospital stay was 6 days (range 5–11 days). The mean blood loss was 350 mL (range 50–2,500 mL). Complications were graded using the Clavien-Dindo scale [13–15]. Donor complications have been divided into postoperative complications (within 90 days) and complications that occurred >90 postoperatively. Donor complications are detailed in Tables 5, 6. 9 (16%) donors experienced complications within 90 days of surgery, and 3 (5%) donors experienced complications 90 days or more post donation surgery. Immediate postoperative complications included three grade one complications–one donor had a symptomatic urinary tract infection and received oral antibiotics, and two donors received antibiotics for wound infections. Two donors experienced grade 3A and 4 donors experienced grade 3B complications within 90 days of surgery (Table 5). Two (3%) donors required drainage of fluid collections via interventional radiology 2 weeks after donation surgery, and one donor presented with a diaphragmatic hernia 7 weeks post donation surgery. One donor required re-laparotomy for a persistent bile leak 6 weeks postoperatively, and one donor required a re-laparotomy day 1 post living liver donation for refixation of the left liver lobe post right lobe donation. One donor developed an incisional hernia 1 month post donation surgery and underwent surgical repair. Three donors experienced complications 90 days or more post their donation surgery (Table 6). One donor developed a diaphragmatic hernia 8 months post liver donation, and two donors required incisional hernia repairs 17 months and 3 years after donation. There were no grade 4 or 5 complications in living liver donors. There was no donor mortality (average follow up 35 months, range 12 weeks–5 years 7 months).

**TABLE 4:** Donor characteristics.

Sex	
Female	36 (66%)
Donation type	
Right lobe	52 (96%)
Left lobe	2 (4%)
Relationship to recipient	
Related directed	35 (65%)
1st degree relative	30 (56%)
2nd degree relative	5 (9%)
Unrelated directed	17 (31%)
Partner	6 (11%)
Friend	6 (11%)
Sister/brother-in-law	4 (7%)
Stepfather	1 (2%)
Unrelated undirected	2 (4%)
Age (years)	33 (18–58)
BMI (kg/m2)	27 (17–31)

**TABLE 5:** Post-operative complications in liver donors (within 90 days).

Clavien-Dindo classification (grade)	No. of complications
1	3
2	0
3A	
Drainage of biloma	2
3B	
Diaphragmatic hernia	1
Incisional hernia	1
Re-laparotomy	2
4A	0
4B	0
5	0

**TABLE 6:** Complications >90 days postoperatively in living liver donors.

Clavien-Dindo classification (grade)	No. of complications
1	0
2	0
3A	
Drainage of biloma	0
3B	
Diaphragmatic hernia	1
Incisional hernia	2
4A	0
4B	0
5	0

### Recipient Outcomes

In total 54 LDLT were performed between January 2019 and August 2024. Recipient characteristics are reported in Table 7. End-stage liver disease secondary to Primary Sclerosing Cholangitis (PSC) and HCC were the most common indications for LDLT in 22 (41%) and 11 (20%) of patients respectively. The median MELD score was 13 (range 6–32). Mean time on the LT WL was 1 year (range of 4 days–15 years, 1 month) and the mean length of hospital stay after transplantation was 21 days (range of 10–52 days).

**TABLE 7:** Recipient characteristics.

Sex	
Female	27 (50%)
Aetiology	
PSC	22
ASH	4
NET	4
MMA	4
NASH	3
AIH/PSC	3
HBV	3
GSD (type 1a and 1b)	2
SBC	2
PBC	2
PFIC type 3	1
HCV	1
Caroli Disease	1
Polycystic liver disease	1
Hemochromatosis	1
HCC (included in above)	11
Age (years)	42 (16–71)
MELD Score	13 (6–32)

PSC, primary sclerosing cholangitis; HCC, hepatocellular carcinoma; MMA, methylmalonic aciduria; GSD, glycogen storage disease; PFIC, primary familial intrahepatic cholestasis; HCV, hepatitis C virus; HBV, hepatitis B virus; ASH, alcoholic liver cirrhosis; NET, neuroendocrine tumour; NASH, non-alcoholic steatohepatitis; SBC, secondary biliary cholangitis; MELD, model for end stage liver disease.

The mean cold ischemia time was 163 min (range 115–290 min), the mean warm ischemia time was 35 min (range 21–55 min), mean OR time 508 min (355–760 min). The mean blood loss was 3.3L (range 0.2–31.5 L), mean actual graft-to-recipient weight ratio (GRWR) 1.08 (range 0.55–1.75). Thirty-five LDLT (65%) recipients had Roux-en-Y reconstructions, 17 (31%) patients had a duct-to-duct biliary anastomosis. One recipient had a combination of duct to duct, and Roux-en-Y biliary anastomosis and one recipient had a duct to duodenum biliary anastomosis. The mean ICU stay was 3.7 days (range 1–40 days).

25 (46%) complications were observed in 22 patients (40%). There were 14 grade 3A, 8 grade 3B, 1 grade 4A, and 2 grade 5 complications as shown in Table 8. Three (5%) recipients developed hepatic artery thrombosis 2-, 5- and 7-day post LDLT. All thromboses were urgently managed in the OR with thrombectomy saving the living liver grafts. Following the thrombectomy in the OR, all hepatic arteries were patent. Biliary complications occurred in 11 patients (bile leaks in 6 (11%) patients and biliary stricture in 5 (9%) patients). All bile leaks occurred within 3 months of the LDLT, 2 out of 4 biliary strictures occurred within 3 months of surgery and the remaining 2 were late onset strictures. Bile duct stenosis was diagnosed based on MRCP findings or recurrent cholangitis, while bile leaks were diagnosed if the bilirubin level in the drain was >3 times the serum bilirubin level. 4 patients with bile leaks were treated conservatively, with the surgical drain remaining in place until the bile leak has resolved. Two patients required percutaneous transhepatic cholangiography drainage for their bile leak. Recipients with biliary stenosis were managed with progressive stenting protocols via endoscopic retrograde cholangiopancreatography, or with percutaneous transhepatic cholangiography drainage. None of the patients with biliary complications required surgical revision of the anastomosis. One recipient who had a hepatic artery thrombosis 7 days post LDLT developed biliary complications and underwent a re-transplantation 16 months later with a deceased donor liver transplant. A second LDLT recipient developed chronic rejection and ischemic type biliary lesions (ITBL). The patient was listed for re transplantation 15 months after LDLT and underwent re-transplantation 2 years after her LDLT. There were two LDLT recipient deaths, neither of whom developed biliary of vascular complications. One recipient had an acute cellular rejection 1 month after LDLT, she was treated for her rejection with rabbit anti-thymocyte globulin (r-ATG). However, she had a severe adverse reaction to r-ATG with a therapy resistant systemic inflammatory reaction, which resulted in resuscitation and transfer to the ICU for extra corporeal membrane oxygenation (ECMO). Unfortunately, she passed away 6 weeks after LDLT. A second recipient was found to have metastatic gallbladder cancer during the LDLT, after the living donor hepatectomy had already been performed. Even retrospectively, this metastatic gallbladder disease could not be visualized on the preoperative scans. He initially recovered well after surgery, but experienced respiratory complications 1 week after LDLT. Due to the poor prognosis, active treatment was withdrawn, and he passed away soon after. There was no further recipient mortality (average follow up 35 months, range 12 weeks–5 years 7 months).

**TABLE 8:** Post-operative complications in transplant recipients.

Clavien-Dindo classification (grade)	No. of complications
3A	
Bile duct stenosis	4
Bile leaks	11
Fluid collection	3
3B	
Hepatic artery thrombosis	3
Post-operative bleeding	2
Incisional hernia repair	2
Re-laparotomy for intra-abdominal abscess	1
4A	
CVVH	1
4B	0
5	2

CVVH, Continuous Veno-Venous Hemofiltration.

## Discussion

This study reports the outcomes of 54 living liver donation and LDLT surgeries performed at Erasmus University Medical Centre between January 2019 and August 2024. It also outlines several key steps essential for the safe and effective implementation–and subsequent expansion–of a LDLT program. The introduction of a LDLT program represents a valuable addition to any existing LT program. It has the potential to enhance access to LT and improve outcomes–particularly relevant given the persistently high wait list delisting and mortality seen in the Netherlands [16].

Ensuring donor safety and minimizing the risk of complications remain the most critical priorities of any live donor program [17]. Institutional experience–including rigorous donor selection processes and comprehensive post donation care–is fundamental to the safety and overall success of LDLT programs [18]. At Erasmus MC, the majority of donations and transplants have involved right lobe grafts, primarily due to the liver volume required by recipients.

Establishing a successful and sustainable LDLT program requires deliberate strategies to address and overcome professional resistance [19]. A persistent concern in many Western countries has been the ethical dilemma of subjecting healthy individuals to the inherent risk of major surgery. However, this resistance tends to diminish when transplant teams are confident that donor safety is prioritized above all else, and when living liver donation is clearly based on informed consent and a deep respect for individual autonomy [19]. At our centre, all donors have expressed satisfaction with their decision to donate and none have reported regret. Donor follows up at Erasmus MC focuses not only on physical recovery, but also emotional and psychological wellbeing. Donors’ quality of life is actively assessed through self-reported questionnaires which donors complete pre donation, 6 weeks, 3 months, 6 months, 12 months and yearly post donation. Notably, we have had not observed major late-term complications aside from incisional hernias. From the inception of the program, a steadfast commitment to the principle “donor safety first” has fostered widespread acceptance and support of living liver donation and LDLT within our institution. With increasing experience, we have gradually expanded the program to include more complex recipient cases, for example, recipients after liver resections, after Whipple procedures, patients with polycystic liver disease and patients with partial portal vein thrombosis. Our growing expertise has also enabled us to perform more technically advanced procedures including domino LDLTs, manage small-for-size syndrome after LDLTs, perform left lobe donations, and reconstruction of segment 5/8 veins in right lobes using PTFE grafts. Looking ahead, we anticipate that we will be able to offer LDLT to increasingly complex recipients, such as patients requiring re-transplantation [20].

LDLT recipient outcomes at our centre have been highly encouraging, with excellent patient and graft survival and an acceptable rate of postoperative complications. LDLT offers a substantial survival benefit to patients with end stage liver disease. Even recipients with MELD scores as low as 11 have an additional 13–17 years of life expectancy compared to similar patients at our centre who did not receive a LDLT [21]. Within the LDLT program at Erasmus MC, the 1-year graft and patient survival after LDLT was 97%. LDLT outcomes typically improve with increasing experience; centres performing less than 20 LDLT annually usually report poorer outcomes [22]. Our high success rate is likely attributed to the significant planning and development that preceded our first LDLT; as well as the strict selection criteria applied to our LDLT recipients.

Despite the high one-year graft and patient survival rates, postoperative complications were observed in 40% of patients within the first 90 days after LDLT (Table 8). This is consistent with existing literature, where complication rates of up to 47% within the first 90 days postoperatively have been reported–most commonly biliary, vascular and haemorrhagic complications [18]. A significant number of recipients underwent hepaticojejunostomies, likely due to the high prevalence of patients with PSC as an indication for LT for whom this technique is routinely employed. While this procedure is also often performed in the DDLT setting at our institution, it is well documented that this approach carries an increased risk of biliary complications [23]. Although limiting donor selection to those with favourable anatomy could potentially reduce the incidence of biliary complications–by increasing the feasibility of duct-to-duct anastomosis–it is challenging to justify excluding otherwise ideal donors based solely on biliary anatomy, especially given the already stringent donor criteria in place.

## Conclusion

We successfully established a LDLT program in the Netherlands, achieving excellent early outcomes for both donors and recipients. One year graft and patient survival was 97% and 97% respectively, and no donor mortality was observed. The number of LDLTs increased annually, reflecting growing confidence and experience among both patients and healthcare providers. The importance of allowing time for all stakeholders to adapt to and gain trust in the LDLT process cannot be overstated. While the establishment of an LDLT program presents many unique challenges, these can be successfully overcome through careful planning, dedication and commitment.

## Data Availability

The original contributions presented in the study are included in the article/supplementary material, further inquiries can be directed to the corresponding author.

## Abbreviations

BMI, body mass index; CT, computer tomography scan; ECG, electrocardiogram; GRWR, graft-to-recipient weight ratio; HCC, hepatocellular carcinoma; ICU, Intensive care unit; LDLT, Living donor liver transplant; LLD, living liver donor; LT, Liver transplant; MELD, model for end stage liver disease; MRCP, magnetic resonance cholangiopancreatography; MRI, magnetic resonance imaging; OR, operating room; PSC, Primary sclerosing cholangitis; WL, Waiting list.
